# Estimating the population size of people who inject drugs in Canada, 2021

**DOI:** 10.14745/ccdr.v51i09a06

**Published:** 2025-10-09

**Authors:** Anson Williams, Justin Sorge, Simone Périnet, Qiuying Yang, Joseph Cox, Matthew Bonn, Ashley Smoke, Nashira Popovic

**Affiliations:** 1 Centre for Communicable Diseases and Infection Control, Public Health Agency of Canada, Ottawa, ON; 2 Department of Epidemiology, Biostatistics and Occupational Health, McGill University, Montréal, QC; 3 Canadian Association of People Who Use Drugs, Dartmouth, NS; 4 Ontario Network of People Who Use Drugs, ON, Canada

**Keywords:** people who inject drugs, injection drug use, HIV, hepatitis C, population size estimates

## Abstract

**Background:**

People who inject drugs are disproportionately affected by HIV and hepatitis C infections. Estimating the size and distribution of this population is essential in monitoring infectious diseases rates and progress towards elimination.

**Objective:**

This study aims to estimate the population sizes of people in Canada who have ever injected drugs, stratified by sex (assigned at birth), province/region and steroid injection, and those who have recently injected drugs (past 12 months), stratified by sex and steroid injection. While a previous national study reported estimates of recent injection by province, this study provides the first estimates of people who have ever injected drugs at both the national and provincial/regional levels. It is also the first to incorporate stratification by sex and steroid injection, using the most currently available data.

**Methods:**

Using combined cycles (2017–2021) of the Canadian Community Health Survey (CCHS), a nationally representative population-based survey, we applied the weighted prevalence of injection drug use to the 2021 Statistics Canada national population size estimate of individuals aged 15 years or more. To this, further adjustments were made using additional data to account for populations not sampled in the CCHS and under-reporting of injection drug use in surveys.

**Results:**

In 2021, an estimated 388,400 (95% CI: 338,900–436,500) people in Canada had ever injected drugs, representing 1.22% of the Canadian population 15 years of age and older. Among these, 75% were male and 25% were female. These estimates varied across regions, ranging from 0.92% to 2.47%. The estimated number of people who have recently injected drugs was 100,300 (95% CI: 82,300–119,200) or 0.31% of the population, of which 74% were male and 26% were female.

**Conclusion:**

Estimates of people who inject drugs at the national and provincial/regional levels can be used to track key epidemiological metrics that inform public health policy and programming.

## Introduction

In Canada, people who inject drugs face a disproportionate burden of sexually transmitted and blood-borne infections (STBBI), including HIV and hepatitis C, due to intersecting risk factors that increase their vulnerability to STBBI transmission ((1)). In 2022, 24.5% of the 1,848 estimated new HIV infections occurred among people who inject drugs, an increase from 22.2% of the 1,610 new infections in 2020 ((2)). Regarding hepatitis C, it was estimated that in 2021, 36.9% of people who had recently injected drugs (in the past 6−12 months) had chronic hepatitis C ((3)).

Accurate estimates of the population size of people who inject drugs are crucial for planning resource allocation and informing harm reduction policies and programs, since injection drug use (IDU), particularly through sharing of injection equipment, increases the risk of transmission of blood-borne infections ((4)). From an epidemiological perspective, population size estimates help quantify burden of disease, monitor trends and measure progress towards elimination targets (5,6). Various methods exist to produce population size estimates, each requiring unique data sources, impacting the feasibility and validity of the estimates ((7)).

In Canada, national estimates of people who inject drugs were published using indirect multiplier methods ((8,9)), while provincial and local estimates have used administrative health data linkage ((10)) and capture-recapture methods ((11)). The use of population-based surveys is a method previously employed for estimating the population size of people who inject drugs ((12,13)). This method uses the proportion of people who inject drugs (i.e., self-reported information) within a given population and multiplies it by the total population size of the respective jurisdiction ((14)). This approach is feasible nationally, as it utilizes existing and representative data sources; however, limitations exist when adjustments are not made to account for unsampled populations within surveys, and under-reporting of the behaviours of interest. This study aims to estimate the population size of both people who have ever injected drugs, and who have recently (in the past 12 months) injected drugs for 2021 by sex and province/region, by implementing an adjusted direct multiplier method using recent national survey data and additional data to account for unsampled populations.

## Methods

A crude portion of the estimate was produced using data from Statistics Canada’s Canadian Community Health Survey (CCHS), otherwise referred to as a CCHS-derived estimate. The CCHS is a nationally representative cross-sectional, population-based survey with ~97% coverage of the Canadian population, described elsewhere ((15)). Coverage of the CCHS excludes persons living in Indigenous communities, full-time members of the Canadian Forces, institutionalized populations, children aged 12–17 that are living in foster care, and persons living in the Québec health regions of Nunavik and Terres-Cries-de-la-Baie-James. For this analysis, CCHS 2017–2021 data were combined using the pooled approach to combining CCHS cycles, noting that data from each province and territory were not captured in every cycle ((16)). The CCHS asks participants about the use of various substances, routes of administration, and recency of use. For this analysis, weighted proportions of both people who have ever injected drugs and who have recently injected drugs were calculated to account for survey design. These weighted proportions were applied to Statistics Canada’s 2021 population aged 15 years and older ((17)). Weighted estimation and bootstrap variance were used to calculate CCHS model inputs and 95% confidence intervals (CIs) using the PROC SURVEYFREQ procedure. Analyses were performed using SAS EG version 7.1 ((18)).

In addition to the CCHS-derived estimate, four additional estimates were computed for populations that were not captured in the CCHS sampling frame. First, an estimate of people who inject drugs among First Nations peoples living in First Nations communities was made by applying IDU data from a cross-sectional biobehavioural survey implemented by First Nations in Saskatchewan and Alberta (19) to corresponding population size estimates from Statistics Canada ((17)). Second, an estimate of people who inject drugs among people who are incarcerated was made by applying IDU data from Correctional Services Canada ((20,21)) to population size estimates from Statistics Canada (22). Only people incarcerated in federal prisons were included in this adjustment, as people serving provincial sentences of less than two years would have been eligible to be sampled by the CCHS. Third, the number of people who inject drugs among active military personnel was estimated, however, due to an absence of data on IDU in the military, the proportions of IDU were assumed to be the same as the CCHS. These proportions were then applied to population size estimates from the Canadian Armed Forces ((23)). Lastly, the number of people who inject drugs experiencing homelessness or unstable housing was estimated. Data from the Tracks survey of people who inject drugs were used and the proportion of people who inject drugs reporting homelessness and/or unstable housing within the past six months was applied to the CCHS-derived estimate of people who have recently injected drugs ((1)). This adjustment applied only to estimates of recent injection because only individuals who had injected drugs six months prior to recruitment are included in the Tracks survey, and individuals experiencing unstable housing beyond this timeframe would be eligible to be sampled by the CCHS. After each unsampled group was estimated, they were added to the estimates derived from the CCHS to form the main estimates of people who have ever injected drugs, and who have recently injected drugs. Since all data sources involved self-reported IDU behaviours, a final adjustment to the main estimates was made to account for underreporting.

For this adjustment, the weighted sensitivity of self-reported substance use of injectable substances compared to a gold standard laboratory detection test in hair samples, taken from a meta-analysis, was used ((24)). The weighted sensitivity was calculated by assigning each study a weight proportional to its sample size when combining results. This weighted sensitivity (52.35%) was applied to the main estimate to derive a final estimate of people who inject drugs. A diagram of the method is presented in the **Appendix** Supplementary Figure S1.

A 95% CI was used to produce plausible ranges around each estimate and were obtained using original data sources, where available. The 95% CIs were not available for both people who have ever, and recently, injected drugs among people living in First Nations communities, people who are incarcerated, and those experiencing unstable housing among people who inject drugs. In these situations, 95% CIs were constructed using parametric bootstraps with 1,000 simulations of N samples of n/N probability from the binomial distribution and subsequently removing the upper and lower 2.5 percentiles ((25−27)).

Estimates were stratified by sex (assigned at birth) for both people who have ever, and recently, injected drugs and by geographic region for people who have ever injected drugs. Due to insufficient observations in smaller provinces, estimates for each individual province could not be produced; therefore, some were grouped into larger geographic regions. Estimates over 1,000 were rounded to the nearest 100, and those under 1,000 to the nearest 10. These analyses were conducted in Microsoft Excel, with data inputs presented in Supplementary Tables S1–S6.

### Sensitivity analysis: Effect of including people who inject steroids

A sensitivity analysis was conducted to assess the impact of excluding individuals who reported injecting only steroids on the CCHS. People who inject steroids represent a unique subset of people who inject drugs, and previous literature has suggested that these individuals should be distinguished from people who inject other substances, due to distinct differences in lifestyle and injecting practices ((28,29)). This adjustment was applied to the CCHS-derived estimate by removing individuals who exclusively injected steroids from the survey responses. Results are presented under both scenarios.

## Results

In 2021, an estimated 388,400 (95% CI: 338,900–436,500) people in Canada had ever injected drugs, representing 1.22% of the population aged 15 and older (**Table 1**). Of these, approximately 75% were male (n=290,800) and 25% female (n=97,500). For those who have recently injected drugs, the estimated prevalence was 100,300 (95% CI: 82,300–119,200) people, or 0.31% of the population aged 15 and older. Similarly, 74% were male (n=74,600) and 26% were female (n=25,600). When excluding individuals who injected only steroids, the prevalence of people who have ever injected drugs decreased by 9.83% to 350,200 (95% CI: 317,200–381,800), and people who have recently injected drugs decreased by 0.60% to 99,700 (95% CI: 81,900–118,600). These reductions were observed only among males, as no female respondents reported injecting only steroids.

**Table 1:** National population size estimates of people who inject drugs by sex (assigned at birth), Canada, 2021

Estimate		Population sizes of people who inject drugs in Canada	
		% (Plausible range)	n (Plausible range)
Including steroid-only injection	People who have ever injected drugs	1.22 (1.06–1.37)	388,400 (338,900–436,500)
	Male	1.84 (1.62–2.05)	290,800 (256,500–323,700)
	Female	0.61 (0.51–0.70)	97,500 (82,300–112,700)
	People who have recently injected drugs (past 12 months)	0.31^a^ (0.26–0.37)	100,300^a^ (82,300–119,200)
	Male	0.47^a^ (0.39–0.56)	74,600^a^ (61,900–88,000)
	Female	0.16^a^ (0.13–0.19)	25,600^a^ (20,300–31,200)
Excluding steroid-only injection	People who have ever injected drugs	1.10^a^ (0.99–1.20)	350,200^a^ (317,200–381,800)
	Male	1.60^a^ (1.48–1.71)	252,600^a^ (234,600–269,600)
	Female	0.61^a^ (0.51–0.70)	97,600^a^ (82,600–112,000)
	People who have recently injected drugs (past 12 months)	0.31^a^ (0.26–0.37)	99,700^a^ (81,900–118,600)
	Male	0.47^a^ (0.39–0.55)	74,000^a^ (61,400–87,300)
	Female	0.16^a^ (0.13–0.19)	25,600^a^ (20,300–31,200)

^a^ Estimates have a high level of sampling variability (15.0< coefficient of variation <35.0). These data should be interpreted with caution

**Figure 1:**
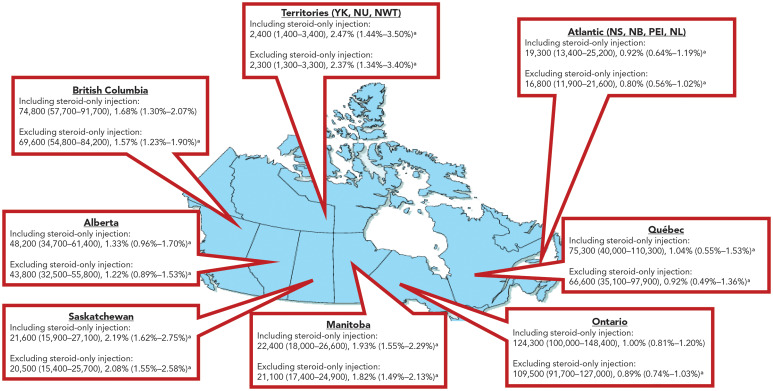
Regional population size estimates of people who have ever injected drugs, Canada, 2021^a^ Abbreviations: NB, New Brunswick; NL, Newfoundland and Labrador; NS, Nova Scotia; NU, Nunavut; NWT, Northwest Territories; PEI, Prince Edward Island; YK, Yukon ^a^ Estimates have a high level of sampling variability (15.0< coefficient of variation <35.0). These data should be interpreted with caution

When stratified by region, some geographic variation across Canada was observed (**Figure 1**). The highest prevalence of people who have ever injected drugs was estimated in the territories at 2.47%, although this represents the smallest estimated number of people at 2,400 (95% CI: 1,400–3,400). In comparison, the Atlantic region had the lowest estimated prevalence of 0.92%. The province with the highest estimated number of people who have ever injected drugs was Ontario with 124,300 (95% CI: 100,000–148,400), translating to 1.00% of the adult population. When broken down into groups not sampled in the CCHS (**Table 2**), the highest prevalence was observed among people who are incarcerated, with 21.75% reporting having ever injected drugs, and 10.80% reporting having recently injected drugs. The highest estimated number of people who have recently injected drugs was observed among people experiencing homelessness or unstable housing at 20,300, however, it was not possible to calculate a prevalence rate due to the lack of a denominator. In comparison, an estimated 16.7% of people living in First Nations communities had ever injected drugs, with 8.1% reporting recent injection. Active military personnel were estimated to have the lowest prevalence, with an estimated 0.51% reporting having ever injected drugs, and 0.04% reporting having recently injected drugs. Estimates of model inputs are presented in the Appendix, Supplementary Tables S1–S6.

**Table 2:** National population size estimates of people who inject drugs among additional populations with data sources (includes steroid-only injection), Canada, 2021

Population	Estimates of people who have ever injected drugs^a^		Estimates of people who have recently injected drugs^a^		References
	% (Plausible range)	n (Plausible range)	% (Plausible range)	n (Plausible range)	
CCHS-derived estimate (Canadian population aged 15+)	0.51 (0.44–0.58)	161,800 (140,300–183,300)	0.04^b^ (0.02–0.05)	12,200^b^ (7,500–16,800)	((15,17))
People living in First Nations communities^c^	16.7 (14.9–18.2)	38,000 (34,000–41,500)	8.1 (6.9–9.4)	18,500 (15,700–21,400)	((17,19)
People who are incarcerated	21.7 (20.0–23.2)	3,000 (2,800–3,300)	10.80 (6.64–14.96)	1,500 (930–1,940)	((20−22))
People experiencing homelessness or unstable housing^d^	N/A	N/A	N/A	20,300 (18,900–22,200)	((1))
Active members of the Canadian Armed Forces^e^	0.51 (0.44–0.58)	370 (320–410)	0.04 (0.02–0.05)	30 (20–40)	((23))

Abbreviations: CCHS, Canadian Community Health Survey; N/A, not applicable

^a^ Results in this table are shown before the adjustment for false negative self-reporting of injection drug use (IDU), and include people who inject steroids only

^b^ Estimates have a high level of sampling variability (15.0< coefficient of variation <35.0). These data should be interpreted with caution

^c^ Census data were available from 2021, however, data collection issues led to a higher number of incompletely enumerated reserves and settlements and a lower estimated number of people living on reserve compared to the 2016 census (Statistics Canada, 2022)

^d^ Data on IDU among people experiencing homelessness and/or unstable housing was not available. Instead, the proportion of people who inject drugs reporting unstable housing within the past six months was applied to the baseline estimate of people who inject drugs from the CCHS

^e^ Due to an absence of data on IDU in the military, the prevalence of people who inject drugs was assumed to be the same as the general population

## Discussion

This study used an adjusted direct multiplier method, combining data from national population-based surveys with additional data sources, to estimate the number of people who inject drugs in Canada in 2021. Potential response bias in survey data was also accounted for. As a result, it is estimated that there were 388,400 people who have ever injected drugs and 100,300 who have recently injected drugs. When interpreting these estimates, it is important to consider the broader social and historical contexts that affect people who inject drugs. Inequities in the social determinants of health, as well as factors such as intergenerational trauma, socioeconomic disparities, and the impacts of colonialism and institutional racism are deeply embedded within the experiences of people who inject drugs (30,31). These underlying factors are difficult to measure and incorporate into an estimation method such the one used in this study.

Previously published estimates in the United States, using comparable methodologies, provide similar estimates of recent IDU, with one study reporting 0.30% (95% CI: 0.19%–0.41%) ((13)) and another reporting a range of 0.24% to 0.59% ((12)). Previously published estimates in Canada vary due to differences in methodology. A study by Jacka *et al.* (2020) used provincial data on recipients of Opioid Agonist Therapy (OAT) and the proportion of people who inject drugs who received OAT to estimate the population size in 2011 and modelled annual increases up to 2016 using data from two provinces. For 2016, they obtained an estimate of 0.70% (range: 0.62%–0.78%) or 171,900 people aged 15 to 64 years who recently injected drugs (8). This estimate is higher than our most comparable estimate of recent IDU for the year 2021 at 0.31% (95% CI: 0.26%–0.37%). This difference might be explained by a sub-optimal sampling of the target population using our data sources and the use of modelling by Jacka *et al.* to project the population size using older data sources. However, we cannot exclude the impacts of the opioid and toxic drug supply crisis, which would not have been accounted for by Jacka *et al.* due to the reference period of their estimate. Janjua *et al.* (2018) estimated that 41,358 (95% CI: 40,944–41,771) people in British Columbia had recently (defined as in the past three years) injected drugs during the period 2013–2015, using an algorithm based on diagnostic codes and prescriptions records in healthcare administrative datasets. Due to major differences in reference periods, our provincial lifetime injection estimate should not be compared to this estimate.

When comparing to other estimates ((8)), the estimates in the current study suggest a potential decrease in the number of people who inject drugs in Canada, which may be attributed to differences in methodologies with previous estimates, but may also be reflective of broader trends related to IDU. Notably, the estimates in this study are the first to partly capture some of the impacts of the COVID-19 pandemic, within the context of the ongoing opioid crisis. The pandemic worsened substance-related harms due to reduced access to services, increased solitary drug use, lack of assisted injections, and sharing or reusing supplies ((32)). Between 2016 and 2023, there were 44,592 reported opioid toxicity deaths in Canada ((33)). Although not all opioid toxicity deaths are attributed to IDU, mortality among people who use drugs in the years following the last published Canadian estimate is likely an important factor in the observed reduction in the population size of people who inject drugs. Another potential contributing factor is recent data suggesting a shift away from injection as the primary mode of consumption in some provinces. In British Columbia, injection was the leading mode of consumption in drug toxicity deaths in 2016, but by 2021, smoking was reported in 56% of deaths compared to 20% for injection ((34)). Similar trends were observed in Ontario, where deaths with indication of injection alone dropped by 64.4%, from 29% in 2017 to 10.3% in 2021, while inhalation-related deaths rose from 22% to 43.5% ((35)). Although drug toxicity deaths are not a direct reflection of all drug use behaviours, these data may suggest a downward trend in injection in these large provinces.

A primary strength of the estimation method used for the current study is the use of the most currently available data sources, which cover the beginning of the COVID-19 pandemic and the ongoing opioid crisis. Another strength is the replicability of this estimation method, allowing the 2021 estimates to serve as an initial data point, which can be repeated as new data becomes available to observe trends in the population of people who inject drugs. While a previous study has reported estimates of people who have recently injected drugs by province, the current study is the first to provide national and provincial/regional estimates of people who have ever injected drugs and to incorporate stratification by sex and steroid-only injection. Another strength of this method is the attempt to account for response bias, for which survey data can be particularly vulnerable. Due to the nature of questions being asked, survey respondents may be hesitant to disclose substance use behaviours due to stigma and discrimination, as well as fear of legal repercussions, among other reasons ((7,24,36)). Failure to account for this bias would likely have led to an underestimation of people who inject drugs.

## Limitations

There are several limitations to the methods used in this study, mainly related to the availability and generalizability of data sources. First, people who inject drugs may not be well represented in the sampling of government surveys such as the CCHS, since they may be hard to reach or reluctance to participate ((11,37,38)), leading to uncertainty in the final estimates. Second, there is a potential that people who are incarcerated in provincial prisons may be underrepresented in the CCHS sample, as the timing of their incarceration may limit the likelihood of their inclusion during the sampling period. Third, the survey used to estimate people who inject drugs among those living in First Nations communities is limited to seven communities in Alberta and Saskatchewan and may not be representative of all First Nations communities in Canada, which affects external validity of this estimate. Fourth, CCHS data collection in the territories was limited in the observed cycles of the CCHS, which could potentially affect generalizability of the territorial estimate. However, a sensitivity analysis using territorial data from previous cycles of the CCHS yielded statistically similar results. Fifth, the survey used to estimate the number of people who inject drugs experiencing homelessness or unstable housing excluded Toronto and Vancouver; however, previous phases of the same survey that included these cities showed similar rates of unstable housing, suggesting a minimal impact. Sixth, when excluding people who inject steroids only, regional estimates from the CCHS were not reliable due to insufficient statistical power. Instead, national proportions were used, which has potential to mask regional differences. Seventh, data on IDU among members of the Canadian Armed Forces were not available, and our estimates assume that the level of IDU among military personnel is the same as in the CCHS. Lastly, although CCHS cycles spanning up to five years were used, data were pooled to reach sufficient sample size for reliable estimation. Furthermore, other data sources used were restricted to single-year estimates, precluding estimation at different timepoints. Further detail on limitations and their potential effects on the estimates are outlined in **Table A1**.

## Conclusion

In Canada, people who inject drugs face a disproportionate burden of STBBIs, due to intersecting risk factors such as stigma, discrimination, increased levels of poverty and marginalization, unstable housing, and incarceration history ((1)). Estimating the population size of this group is essential for tracking key epidemiological metrics that inform public health policy and programming. The estimates from this study will serve as a benchmark, to be updated and refined as new data emerges.

While these estimates provide valuable insights, there is a need for further efforts to estimate the broader population of people who use drugs, not only those who inject. Expanding the scope of research to include qualitative data on broader social and historical contexts will provide a more comprehensive understanding of the community.

## Appendix

**Table A1:** Key limitations in the data used to estimate the population size of people who inject drugs in Canada, 2021, and their potential effects

Limitations of data	Potential effect on estimates
Representation of people who inject drugs within CCHS may be low, given they may be hard to reach or reluctant to participate in a government survey.	Underestimation
The CCHS questions regarding injection drug use pertain exclusively to the injection of substances not prescribed by a doctor. Consequently, the data excludes individuals who inject prescription medications for reasons outside of the intended medical purpose. We assess this number of individuals to be small, resulting in minor impact on estimations.	Minimal
Individuals that reside in rural communities may be underrepresented in the Tracks survey among people who inject drugs, which was used to estimate the proportion of people who inject drugs experiencing unstable housing. Individuals residing in rural areas often face increased barriers to accessing services and may be therefore less likely to participate in a survey.	Underestimation
People incarcerated in provincial prisons may be underrepresented in the CCHS sample, as the timing of their incarceration may reduce the likelihood of inclusion within the sampling period.	Underestimation
In the absence of specific data on regular members of the Canadian Armed Forces, we assumed the proportions of IDU among active military personnel were the same as the CCHS sample. Survey data in the United States suggests that illicit drug use among active military members and military veterans differs from the civilian population ((39)).	Unknown
In the adjustment for underreporting of injection drug use behaviours, we assumed the same degree of underreporting across all surveys. Self-report bias is likely to vary depending on the context in which respondents are asked.	Unknown
The data used to estimate the population of people who inject drugs among First Nations Peoples living in First Nations communities was limited to communities in Alberta and Saskatchewan. These data may not be generalizable to all First Nations communities across Canada.	Unknown
Surveys used to estimate the population of people experiencing homelessness or unstable housing are venue-based (i.e., used non-probability-based sampling). As a result, the findings from these surveys may not be representative of all these groups at any given site or across Canada.	Unknown
Regional estimates of people who inject steroids from the CCHS were not reliable due to insufficient observations. Instead, the national proportions of steroid-only injection were used for each region.	Unknown
The biobehavioural survey used to estimate people who inject drugs experiencing homelessness or unstable housing excluded major Canadian cities of Toronto and Vancouver. Previous phases of this survey that did include these cities were also examined, and they reported similar rates of unstable housing.	Minimal

Abbreviations: CCHS, Canadian Community Health Survey; IDU, injection drug use

Supplemental material is available upon request to the author: stbbi.estimates.field.surv-itss.estimations.surv.terrain@phac-aspc.gc.ca

Figure S1: Data sources used to estimate the population size of people who inject drugs in Canada, 2021

Table S1: Data inputs for estimates of people who have ever injected drugs (excluding people who inject steroids only)

Table S2: Data inputs for estimates of people who have ever injected drugs (including people who inject steroids only)

Table S3: Data inputs for estimates of people who have recently injected drugs (excluding people who inject steroids only)

Table S4: Data inputs for estimates of people with a recent history of injection drug use (including people who inject steroids only)

Table S5: Unadjusted Canadian Community Health Survey (CCHS)-derived provincial/regional prevalence of people who have ever injected drugs (including people who inject steroids only)

Table S6: Unadjusted Canadian Community Health Survey (CCHS)-derived national-level prevalence of people who have ever injected drugs and people who have recently injected drugs, by sex (including people who inject steroids only)
